# The current role of renal biopsy in the management of localized renal tumors

**DOI:** 10.4103/0970-1591.57926

**Published:** 2009

**Authors:** Gagan Gautam, Kevin C. Zorn

**Affiliations:** Section of Urology, Department of Surgery, University of Chicago, IL, USA

**Keywords:** Fine-needle aspiration cytology, nephron-sparing surgery, renal biopsy, renal tumor, renal cell carcinoma

## Abstract

**Introduction:**

In the current era of nephron-sparing surgery (NSS) for localized tumors, pretreatment tissue biopsy is being revisited and re-evaluated. Whether a renal biopsy can supplement imaging investigations to change patient management is a subject of current research.

**Materials and Methods:**

We performed a database search in PubMed for manuscripts from 1988 to 2008 using the appropriate keywords. Manuscripts were selected according to their relevance to the current topic and incorporated into this review.

**Results:**

Preoperative renal biopsy has been utilized to effectively distinguish between benign and malignant tumors localized to the kidney with minimal additional morbidity attributable to the procedure. Tissue diagnosis can also potentially grade renal tumors and uncover unusual malignancies. Although its acceptance remains limited, with fear of false negative results, bleeding and tumor seeding, its ability to influence management decisions has been demonstrated in literature.

**Conclusions:**

The role of preoperative renal biopsy for localized renal tumors is likely to increase rapidly in the coming times. With the expanding scope and utilization of NSS, this diagnostic modality will find increased applicability and acceptance in individualizing management protocols in the future.

## INTRODUCTION

With the advent of newer imaging modalities and their widespread application for specific and nonspecific complaints, there has been an increase in the diagnosis of incidentally detected small renal tumors.[1,2] Earlier discovery of these lesions has allowed the application of more minimally invasive therapies, particularly nephron-sparing surgery (NSS) (laparoscopic or robot-assisted partial nephrectomy) and ablative technology (cryoablation and radiofrequency ablation).[3]

The role of pretreatment histological (percutaneous renal biopsy) or cytological (fine-needle aspiration (FNA)) analysis of the small renal mass has continuously evolved during the recent years. After being largely disregarded, renal biopsy has found recent acceptance in selected clinical situations and is now being increasingly used for the evaluation of small renal masses (SRM) <4 cm in diameter.

## METHODS

We performed a database search in PubMed for manuscripts from 1988 to 2008 using the following keywords: “renal biopsy”, “renal fine-needle aspiration cytology”, “nephron-sparing surgery” and “small renal tumors”. Manuscripts were selected according to their relevance to the current topic and incorporated into this review. Details pertaining to biopsy accuracy for diagnosis - particularly histological subtype and tumor grade, false negative rate and complications were collected.

## CURRENT ROLE OF RENAL BIOPSY

Historically, renal core-biopsy or, to a lesser degree, fine-needle aspiration cytology (FNAC) has been used to establish a tissue diagnosis in a renal mass with metastases, an unresectable renal mass, a patient unfit for surgery or indeterminate diagnosis with a suspicion of either an infectious process or an unusual malignancy (e.g., lymphoma, renal capsular sarcoma).[4,5] The role of renal biopsy in localized resectable renal tumors has been curtailed to a large extent with most surgeons basing their management protocols on findings of an enhancing mass determined by axial imaging.

Imaging modalities however have a limited accuracy in the diagnosis, characterization and prognosis of a localized SRM. Dechet *et al*. observed a sensitivity of 74% and a specificity of 20% for the CT scan in the evaluation and characterization of SRMs in comparison to permanent sectioning of the histopathological specimen.[6] Cross-sectional imaging is effective in diagnosing the presence of a SRM but is unable to differentiate a malignancy from a benign tumor (e.g., oncocytoma) in a large proportion of cases.[7] Moreover, subsequent tumor behavior can be more accurately assessed on histopathological determination and grading (obtained on biopsy), especially in ablative or active surveillance protocols. In view of these shortcomings, there has been a resurgence of interest in renal biopsy in relation to localized renal tumors presenting in a variety of clinical scenarios.

## BENIGN VERSUS MALIGNANT TUMORS

Currently, the standard of care for SRMs is open or laparoscopic partial nephrectomy. However, a large proportion of these tumors subjected to surgery are found to be benign in the final histopathology. Frank *et al*. reported benign pathology in 30% of SRMs treated by radical or open partial nephrectomy at the Mayo clinic.[8] Link *et al*. similarly reported a 33.6% rate of detection of benign disease in tumors removed by partial nephrectomy.[9] Other authors have reported a lower incidence of benign tumors, in the range of 19%–26%, among T1a lesions removed by surgery.[10,11]

Owing to these findings, there is a recent concern regarding the large number of unnecessary surgeries being performed in patients, who could otherwise have been safely observed. This has led to an increased interest in pretreatment biopsy with the aim of differentiating benign from malignant tumors. Vasudevan *et al*. retrospectively reviewed data on core biopsies performed for incidental renal masses over a 5-year period. Among the 100 biopsies performed on 92 patients, 70 were considered diagnostic. These diagnostic biopsies could effectively distinguish malignant from benign pathology with a 100% sensitivity and specificity in comparison to the nephrectomy specimen.[12]

The importance of this technique lies in its potential to decrease the number of nephrectomies (radical or partial) inadvertently being performed for benign tumors. Using a policy of routine biopsy for tumors <4 cm, Neuzillet *et al*. could avoid surgery in 15.9% of such patients.[13] On follow-up, no cancer was detected in these patients after the diagnosis of a benign tumor on preoperative biopsy. Similarly, surgery could be avoided in 44% of patients in another series after accurate characterization of the tumor by preoperative biopsy.[14] In a recent retrospective analysis of 152 renal biopsies, malignancy was detected in 56% and benign disease was confirmed in 40% patients. A nondiagnostic biopsy was only observed in 4%. As such, sensitivity for malignancy was 97.7% with a specificity of 100%. Positive and negative predictive value of renal mass biopsy was 100% for both. In their series, at least 60.5% biopsy results significantly impacted clinical management and influenced decision-making with regard to offering the patient conservative management vis-a-vis a therapeutic modality.[15] Although the current standard of care for enhancing, small, solid renal masses does not involve routine use of preoperative biopsy, its use is likely to increase in light of new evidence supporting its accurate role for distinguishing malignant from benign tumors in this clinical scenario.

## GRADING OF RENAL CELL CARCINOMA

Renal cell carcinoma (RCC) encompasses a wide spectrum of disease processes with its clinical behavior and outcome varying on the basis of disease stage and grade. Nuclear grading is considered an important predictor of survival in RCC.[16,17] The incidence of metastases also progressively increases with increasing tumor grade.[18] Most of the incidentally detected renal masses are small, low grade tumors, which grow slowly and have a low metastatic potential.[19] The majority of these tumors are amenable to NSS and may be effectively treated by partial nephrectomy or other ablative techniques. For tumors that are larger than 4 cm in size, preoperative information regarding the grade of the tumor may facilitate management decisions with regard to choosing between NSS and radical nephrectomy. Al Nazer *et al*. performed preoperative FNA on 18 patients undergoing nephrectomy for suspected RCC and graded them from grade I to IV on the basis of cellularity, nuclear to cytoplasmic (N/C) ratios, nuclear pleomorphism and the presence of naked nuclei and prominent nucleoli. They found a 100% correlation with the Fuhrman's grading system performed on the nephrectomy specimens when the tumors were classified as low or high grade and a 72.2% correlation when the tumors were compared grade for grade with the Fuhrman's system.[20] Similarly, in a recent series of 100 renal biopsies, Volpe *et al*. reported an 84% diagnostic rate with 100% histopathological concordance in patients undergoing subsequent nephrectomy. Moreover, RCC subtyping and grading was possible in 93% and 68% of the biopsies with a concordance rate of 100% and 66% respectively.[21]

Further evaluation and establishment of tumor grading on preoperative FNA or percutaneous biopsy may in the near future, make it easier to offer appropriate individualized management strategies to patients with similar imaging findings. It is likely that increasing numbers of larger tumors would come under consideration for NSS without compromising patient outcomes.

## UNUSUAL RENAL MASSES

With a recent increase in the use of preoperative renal biopsy, many unusual and rare renal tumors have been assessed cytologically and histologically. Many variants of sarcomas arising from the kidney or its capsule have been studied and characterized by preoperative FNA.[5,22–24] Johnson *et al*. evaluated the cytological features of primary renal angiosarcoma and correlated them to the final histopathology.[24] Similarly, Silverman *et al*. diagnosed and characterized a low-grade fibromyxoid sarcoma arising from the renal capsule by performing a preoperative FNA and correlated the findings to the nephrectomy specimen.[5]

Uncommon variants of RCC have also been diagnosed and evaluated preoperatively through renal biopsy techniques.[25–27] Sarode *et al*. and Ono *et al*. reported the distinctive features of collecting duct carcinoma on preoperative renal FNA, while Salamanca *et al*. recently elucidated the FNA findings of chromophobe RCC.[25–27] Similar reports characterizing the features of benign tumors such as angiomyolipoma and oncocytoma have also emerged in literature recently and have greatly increased the current knowledge regarding preoperative cytological diagnosis of renal masses.

With an increased understanding of the distinctive features of various renal masses, benign and malignant, an experienced renal pathologist can often define the exact diagnosis preoperatively, thereby greatly facilitating an optimum strategy for subsequent patient management.[28,29]

## ACTIVE SURVEILLANCE PROTOCOLS

Active surveillance for small renal tumors is now considered a viable treatment option in selected patients.[30,31] Slow growth rates of small renal masses and a low metastatic potential has prompted some experts to routinely consider watchful waiting as a management protocol in elderly patients (>75 years) and in patients with considerable comorbidities.[30] Moreover, a delayed intervention has been shown not to result in any significant disadvantage to the patient in terms of subsequent upstaging at the time of the subsequent surgical intervention.[31] In light of this recent evidence, some authors have recommended that active surveillance should be considered a frontline option for renal tumors of size <3 cm and sometimes even in tumors of size up to 5 cm.[3] This shift in policy toward less aggressive modes of management and active surveillance protocols can be greatly facilitated by advances in biopsy techniques and interpretation. It can enable less aggressive tumors to be safely observed in a large proportion of cases and avoid the inherent morbidities associated with a surgical intervention.

## ABLATIVE TREATMENT PROTOCOLS

Percutaneous and laparoscopic ablative techniques such as RFA and cryoablation have shown promising early and intermediate term results and now form an important part of the surgical armamentarium against localized renal tumors. The major attractions of this form of treatment are their technical ease, lesser morbidity and a lower complication rate. These procedures inherently do not involve removal of a specimen for histopathology and unless specific biopsies are taken beforehand, may result in the ablation of many benign lesions, which could otherwise have been managed conservatively. Moreover, residual or recurrent disease after treatment is found in 11.1% of tumors treated by RFA and 1.8% of tumors treated by cryoablation.[32] Conventionally, this is detected by enhancement of the tumor remnant on follow-up CT scan after treatment with the above modalities. Further management of such a situation can be greatly facilitated with knowledge of the histopathological characteristics of the tumor from a biopsy obtained preoperatively. Evaluation and assessment of the long-term outcomes of these modalities also warrants that this information should be recorded since approximately 25% of the tumors undergoing this type of treatment may actually be benign and would not be prone to recurrence in any case.[33] Thus, as the scope and indications of these ablative treatment protocols evolve, it is likely that preoperative and follow-up renal biopsies will play an increasing role in the near future.

## BIOPSY VERSUS FNA

Although, fine-needle aspiration (FNA) of the renal mass is considered less invasive than percutaneous biopsy techniques, its accuracy and diagnostic yield has not been able to match up to the coaxial biopsy procedure using the 18G core biopsy needle. Schmidbauer *et al*. performed simultaneous FNA and renal biopsy in patients with a mean tumor size of 4 cm. The yield from FNA was insufficient for diagnosis in 11% of patients as compared to 3% in biopsy. Sensitivity for detection of malignancy was 90.6% and 95.2%, while specificity was 100% and 100% for FNA and biopsy, respectively. RCC subtype could be accurately diagnosed in 86% of cases by FNA compared with 91% of cases by biopsy. Similarly, FNA could accurately discern the Fuhrman's grade in only 28% of patients vis a vis 76% in case of renal biopsy.[34]

Hence, while some centers continue to use the FNA technique, most authors suggest the use of the coaxial percutaneous renal biopsy method to enhance the diagnostic yield, sensitivity and specificity of the procedure, thereby facilitating therapeutic decision-making.

## CHOICE OF IMAGING MODALITY

Ultrasonography and CT are the commonly used modalities for guiding renal biopsy or FNA. Although these 2 imaging techniques have not been compared within the purview of a clinical trial, each of these has its own advantages and limitations. While ultrasound has the advantages of easy portability and accessibility, real-time imaging and lesser cost, it is entirely operator dependent with a steep learning curve. Although CT-guided biopsy techniques are easier to master and permit better visualization of internal organs and the needle track, they are relatively more cumbersome, entail greater cost and do not permit real-time visualization of the needle at the time of insertion with the patient outside the gantry.[35]

## PERCEPTIONS IN CLINICAL PRACTICE

In spite of recent advances in the techniques and role of preoperative renal percutaneous biopsy, its acceptance in contemporary urological practice remains limited. In a survey of 272 urologists at the 23rd World Congress of Endourology, Kummerlin *et al*. reported that 55.9% of surgeons never performed a biopsy and 41.8% performed it only in rare clinical circumstances. In fact, there was only 1 (0.5%) respondent who stated that he/she asked for a preoperative biopsy more than half the time.[36] Although this scenario is likely to change in the future, it may take a while before research in this field impacts current clinical practice.

## COMPLICATIONS AND FALLACIES OF RENAL BIOPSY

Pneumothorax, renal bleeding, subcapsular hematomas and pseudoaneurysm formation have all been reported in patients undergoing renal biopsy.[37–39] The overall complication rate however remains acceptably low with most series reporting a <2% incidence and some reporting no complications.[15,40] Routine preoperative assessment of coagulation profile and observation for a few hours after the biopsy is considered sufficient for the safe implementation of the biopsy protocol.

Occasional case reports have described needle tract seeding after renal tumor biopsy.[41] However, most contemporary series with longitudinal follow-up have not encountered this phenomenon and as such this entity, on account of its extreme rarity, is not considered a viable deterrent to renal biopsy by most experts. Some authors have speculated that a coaxial biopsy technique (performed through an outer sheath) may prevent tumor seeding in the tract by preventing multiple passes of the biopsy needle directly through the body wall.[42]

The incidence of nondiagnostic biopsies is often regarded as a potential deterrent to routine renal biopsies for localized renal tumors. However, current studies suggest that with expertise and experience, a vast majority of procedures performed can yield definitive diagnostic information with a high degree of accuracy. In an analysis of 66 biopsies, 79% were considered diagnostic with 98% accuracy.[43] In another large series of 152 biopsies, only 4% of the biopsies were considered nondiagnostic.[15] Thus, with further refinements in technique, it is likely that the yield of nondiagnostic biopsies will progressively decrease, thereby resulting in a greater impact on subsequent patient management.

## CONCLUSIONS

Preoperative percutaneous renal biopsy for localized renal masses remains a controversial procedure with limited acceptability among urological surgeons. Recent advances in techniques and interpretation have, however, greatly increased its diagnostic accuracy and applicability. It is now possible to effectively and accurately discern between benign and malignant neoplasms and even to grade renal tumors with this methodology. With expanding indications of NSS and wider application of ablative procedures, this diagnostic technique is set to play a greater role in management protocols for localized renal masses. Further validation and standardization of both, the biopsy technique as well as its interpretation is required before this modality is widely accepted in contemporary urological practice.
